# Factors influencing uptake of HPV vaccination among girls in Germany

**DOI:** 10.1186/s12889-016-3663-z

**Published:** 2016-09-20

**Authors:** Stefanie Schülein, Katherine J. Taylor, Jochem König, Matthias Claus, Maria Blettner, Stefanie J. Klug

**Affiliations:** 1 Epidemiology, Department of Sport and Health Sciences, Technical University of Munich, Georg-Brauchle-Ring 56 (Campus C), Munich, 80992 Germany; 2 Institute for Medical Biostatistics, Epidemiology and Informatics, University Medical Centre, University of Mainz, Obere Zahlbacher Str. 69, Mainz, 55131 Germany; 3 Institute for Teachers’ Health, University Medical Centre, University of Mainz, Kupferbergterrasse 17-19, Mainz, 55116 Germany

**Keywords:** Human papillomavirus, HPV, Vaccine, Uptake, Germany

## Abstract

**Background:**

Adequate coverage is key to the success of human papillomavirus (HPV) vaccination programmes. There is currently no organised HPV vaccination programme in Germany. The aim of this analysis was to determine HPV vaccine uptake as well as factors associated with uptake in nine to 17 year-old girls in Germany during the first year of vaccine availability.

**Methods:**

This analysis is based on data from the Healthcare Access Panel, an established population-based household panel consisting of 55 000 representative households in Germany who were contacted between September and October 2007. A total of 4 747 households included at least one girl aged nine to 17 years. After reading a description of the HPV vaccine, these girls were asked, “Would you have yourself vaccinated against HPV?” Logistic regression analyses were performed to investigate associations between vaccination status and socio-demographic characteristics of the girls and their mothers.

**Results:**

Of the 4 747 girls in the households who received questionnaires, 2 224 (46.9 %) participated in the study and 1 906 (40.2 %) answered the vaccination question. A total of 17.4 % of the girls were already vaccinated, 61.5 % felt positively about doing so, 4.7 % said they would not be vaccinated, and 16.3 % were not sure. The probability of a girl being vaccinated increased with each additional year of age (Odds Ratio (OR): 1.6, 95 % Confidence Interval (CI) 1.5–1.7). Among the 17 year-old girls, 38.5 % (95 % CI 32.6–44.4 %) had been vaccinated. Having a mother with high education (OR: 1.5, 95 % CI 1.0–2.3) or medium education (OR: 1.5, 95 % CI 1.1–2.1) versus basic education was a significant predictor for having been vaccinated. Similarly, medium (OR: 1.5, 95 % CI 1.0–2.4) versus low SES was significantly associated with having been vaccinated. Our analysis showed that during the first year of HPV vaccine availability in Germany, vaccination uptake was low.

**Conclusions:**

Countries with organised HPV vaccination programmes showed much higher vaccination uptake, even in the first year after programme introduction. If vaccination uptake in Germany is to significantly improve in the future, an organised vaccination programme will need to be introduced.

## Background

HPV infection is causally associated with the development of cervical cancer, which is the fourth most common cancer among women worldwide [1]. In 2012, more than 67 000 women in Europe were diagnosed with cervical cancer and more than 28 000 women died from the disease [1]. Efforts to prevent HPV infection, including HPV vaccination, are an important part of prevention strategies to potentially reduce the risk of cervical cancer [2].

Among the more than 100 types of human papillomavirus (HPV), there are approximately 40 which infect the genital tract [3]. Fifteen HPV subtypes have been classified as high risk for cervical cancer development [4]. In particular, HPV 16 and 18 have been strongly associated with cervical cancer [4, 5]. Among the HPV subtypes classified as low risk, HPV 6 and 11 are associated with causing benign genital warts [4, 6].

In 2006, the European Medicines Agency (EMA) and the U.S. Food and Drug Administration (FDA) approved a quadrivalent HPV vaccine for protection against HPV 6, 11, 16 and 18. This was followed by EMA and FDA approval in 2007 and 2009 respectively for a bivalent vaccine that protects against HPV 16 and 18 only. In Germany, a recommendation was issued by the German Standing Committee on Vaccination (STIKO) in March 2007 to vaccinate girls aged 12–17 [7]. In 2014, this recommendation was revised, stating that girls between the ages of nine and 14 should be vaccinated prior to first sexual activity [8]. The cost of the vaccine for girls in the recommended age range is covered by the German health insurance system, in which membership is largely mandatory. Girls may get vaccinated when they visit a paediatrician, gynecologist or general practitioner. However, in Germany there is no organised vaccination programme and high coverage among the target age groups has yet to be achieved.

In some countries, such as the United Kingdom, Canada and Australia, there are ongoing school-based programmes for HPV vaccine delivery [9]. In other countries, such as the Netherlands, population-based, non-school-based programmes have been started [10]. Vaccine uptake has varied widely between different countries [11].

Studies have identified various reasons for refusing to have oneself or ones child vaccinated. Belief in the safety of vaccines in general, as well as perceived susceptibility to HPV infection or cervical cancer have been shown to correlate with acceptance of the HPV vaccine [12–14]. Age of the parents and region of residence were found to be associated with intention to vaccinate a daughter in a Canadian study [15]. Studies have shown inconsistent findings with regards to the association between household income and HPV vaccination acceptance [16, 17].

This analysis aimed to determine the uptake of the HPV vaccine among girls aged nine to 17 years in Germany and to determine factors associated with uptake. The study was conducted during the first year of vaccine availability in Germany and therefore provides valuable baseline data, which can be compared to future studies analysing vaccine uptake among girls in Germany over time.

## Methods

### Study population

This cross-sectional analysis is based on data from the Healthcare Access Panel, an established population-based household panel consisting of 55 000 representative households in Germany who were contacted between September and October 2007. Households were routinely recruited to the panel and the base sample was evaluated on an ongoing basis. Any underrepresented cells were purposively recruited for to ensure representativeness of the base population. A total of 4 747 households in the panel were identified as including at least one girl aged nine to 17 years. These households received an additional question on HPV vaccination, which was included in the standard panel questionnaire. All questionnaires were mailed to the parents via the regular postal service and parents consented to their daughters filling out the question relating to HPV vaccination within the home environment.

### Questionnaire

The HPV vaccination question was introduced with the following explanatory paragraph:

“A prophylactic vaccine against human papillomavirus (HPV) is now available. The vaccine is supposed to prevent cervical cancer as well as genital warts. For girls between the age of 12 and 17 the cost of the vaccination is covered by health insurance.”

Girls between the ages of nine and 17 were asked: “Would you have yourself vaccinated against HPV?” This question had five response categories: “Already been vaccinated”, “Yes, vaccination will occur”, “Yes, vaccination is a possibility”, “No”, and “I am not sure”.

### Education, income and socio-economic status (SES)

Due to the fact that girls below the age of 18 are usually still attending school and living at home, data on education, net household income and profession included in this analysis were retrieved from the mother’s responses to these three questionnaire items.

SES was determined using an approach developed by Winkler, combining data on education, net household income and profession [18]. The index was then modified to take the number of people living in each household into account [19]. The education, household income and profession variables were each reduced to seven categories, and assigned one to seven points. The resulting scores were summed to give a final score between three and 21. These scores were categorised into three SES levels: low (score < 8.5), medium (score ≥ 8.5 and <14.5), and high SES (score ≥ 14.5). If data were missing for one of the three variables, imputation was used to obtain the mean of the two available variables [20].

Education, income and SES are known to have a significant effect on overall health outcomes. Maternal education and income were therefore also investigated separately in this analysis. With regards to education, pupils in Germany may complete one of three types of secondary education. Gymnasium (12–13 years) allows entry into university, while Realschule is completed after 10 years and Hauptschule after 9 years. These three types of school education were classified as follows in our study: high education, medium education and basic education. Income was similarly classified as high (≥3000€), medium (1500€ to 2999€) or low (<1500€).

### Statistical analysis

Anonymised data on the girls’ age and nationality, the mother’s socio-demographic characteristics as well as the girls’ responses to the vaccination question were available for this analysis. The actual vaccine uptake of girls aged nine to 17 was analyzed. Univariable and multivariable logistic regression analyses were performed to determine factors associated with vaccination uptake. One model included age (as a continuous variable), residence in eastern versus western Germany (reference: western Germany), education (reference: basic education) and income (reference: low income); the second model included the same variables, except that SES (reference: low SES) was included instead of education and income. Due to the fact that the SES variable was constructed from the education, income and profession variables, this necessitated the inclusion of these variables in separate logistic regression models in order to avoid collinearity.

Odds ratios (OR) and 95 % confidence intervals (CI) were reported, and p-values less than 0.05 were considered statistically significant. All analyses were performed by two independent researchers, one using STATA/SE 8.1 (College Station, Texas, USA) for Windows (2003), and the other using SAS 9.1.3 (Cary, North Carolina, USA).

## Results

Among the 4 747 households with a girl aged nine to 17 included in the survey, 2 224 (46.9 %) returned the questionnaire. Since 318 girls had not answered the HPV vaccination question, 1 906 (40.2 %) questionnaires were available for analysis. The age distribution of the study sample ranged from 8.9 % nine year-olds to 14.5 % 17 year-olds (Table 1). Most girls (87.4 %) had German citizenship and resided in western Germany (84.9 %). Concerning school education, 21.4 % of the girls’ mothers had high education, 51.2 % medium education, and 24.2 % basic education. The majority of households were in the medium income (1500€ to 2999€) category (53.7 %). About eight percent had mothers in the high SES category, 73.3 % in the medium SES category, 11 % in the low SES category, and 7.6 % of mothers provided insufficient data to determine SES.

**Table 1:** Socio-demographic characteristics of 1 906 responding girls aged 9 to 17 years and their mothers

Age of girls (years)	n	%
9	170	8.9
10	180	9.4
11	183	9.6
12	198	10.4
13	175	9.2
14	228	12.0
15	218	11.4
16	279	14.6
17	275	14.5
Nationality of girls		
German	1665	87.4
Other	5	0.3
Missing	236	12.4
Region of residence		
Eastern Germany	288	15.1
Western Germany	1618	84.9
Highest school education of mother		
Basic education	461	24.2
Medium education	975	51.2
High education	407	21.4
Missing	63	3.3
Net monthly household income		
< 1500€	402	21.1
1500€ to 2999€	1024	53.7
≥ 3000€	455	23.9
Missing	25	1.3
SES of mother		
Low SES	210	11.0
Medium SES	1397	73.3
High SES	154	8.1
Missing	145	7.6
Total	1906	100

As shown in Table 2, 17.4 % (95 % CI 15.7–19.2 %) of girls aged nine to 17 indicated they had already been vaccinated, 35.4 % (95 % CI 33.3–37.6 %) said that vaccination will occur, and 26.1 % (95 % CI 24.2–28.2 %) said it was a possibility. Only 90 girls (4.7 %, 95 % CI 3.8–5.8 %) indicated that they would not be vaccinated, while 311 (16.3 %, 95 % CI 14.7–18.1 %) were not sure.

**Table 2:** Responses to the question “Would you have yourself vaccinated against HPV?”, which was answered by 1 906 girls aged 9 to 17

Would you have yourself vaccinated against HPV?	Girls (aged 9–17)		
	n	%	95 % CI^a^
Already been vaccinated	332	17.4	15.7 - 19.2
Vaccination will occur	675	35.4	33.3 - 37.6
Vaccination is a possibility	498	26.1	24.2 - 28.2
No	90	4.7	3.8 - 5.8
I am not sure	311	16.3	14.7 - 18.1
Total	1906	100	

^a^CI: Confidence Interval

**Fig. 1:**
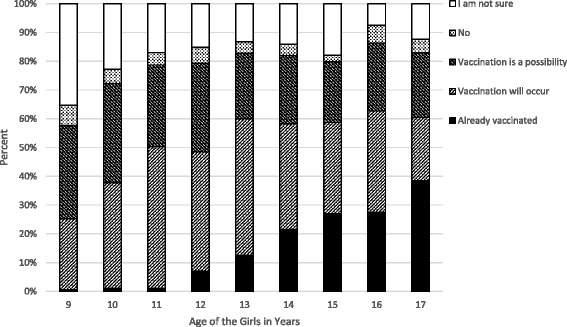
Vaccination status of girls and distribution of responses (%) by age for 1 906 girls

Those who were either already vaccinated or had a positive attitude towards HPV vaccination (‘vaccination will occur’ or ‘vaccination is a possibility’) ranged between 57.7 % (95 % CI 49.8–65.2) (among 9 year-olds) and 86.4 % (95 % CI 81.8–90.2 %) (among 16 year-olds) (Fig. 1). However, among nine, ten and 11 year-olds, who were not covered by the national vaccination recommendation at the time, only 0.6 %, 1.1 % and 1.1 % respectively had been vaccinated. For girls 12 years and older, the proportion of vaccinated girls was considerably larger, and increased with each additional year of age. The 15 and 16 year-olds had similar HPV vaccination uptake (27.1 %, 95 % CI 21.3–33.5 % and 27.6 %, 95 % CI 22.4–33.2 % respectively), while 38.5 % (95 % CI 32.6–44.4 %) of the 17 year-olds had already been vaccinated. The vaccine uptake among the 12 to 17 year-olds was 23.8 % (95 % CI 21.6–26.1 %).

Results of the univariable logistic regression analyses showed that with each increasing year of age, the likelihood of a girl having already been vaccinated increased (OR: 1.6, 95 % CI 1.5–1.7). Girls living in eastern Germany were more likely to have been vaccinated than those living in western Germany (OR: 1.4, 95 % CI 1.0–1.9). Additionally, having a mother with high education (OR: 1.6, 95 % CI 1.1–2.3) or medium education (OR: 1.4, 95 % CI 1.0–1.9) as compared to basic education, was statistically significantly associated with having been vaccinated (Table 3). Girls with mothers who had a high income were also more likely to have been vaccinated (OR: 1.5, 95 % CI 1.0–2.1) than those with a low income.

**Table 3:** Results of Univariable and Multivariable logistic regression analyses for girls already vaccinated versus girls who had not been vaccinated^a^

	Univariable		Multivariable including education^b^		Multivariable including SES^c^	
Variable	OR^j^	95 % CI^k^	OR^j^	95 % CI^k^	OR^j^	95 % CI^k^
Age of the girl^d^	1.6	1.5–1.7	1.6	1.5–1.7	1.6	1.5–1.7
Residence in eastern Germany^e^	1.4	1.0–1.9	1.3	0.9–1.9	1.3	0.9–1.8
High Education (mother)^f^	1.6	1.1–2.3	1.5	1.0–2.3	-	-
Medium Education (mother)^f^	1.4	1.0–1.9	1.5	1.1–2.1	-	-
High Income (mother)^g^	1.5	1.0–2.1	1.3	0.9–2.0	-	-
Medium Income (mother)^g^	1.1	0.8–1.4	1.1	0.8–1.6	-	-
High SES (mother)^h^	1.5	0.9–2.6	-	-	1.7	0.9–3.0
Medium SES (mother)^h^	1.3	0.9–2.0	-	-	1.5	1.0–2.4

^a^Models show the odds ratios for girls indicating ‘Already been vaccinated’ versus ‘Vaccination will occur’, ‘Vaccination is a possibility’, ‘No’ or ‘I am not sure’ for the question ‘Would you have yourself vaccinated against HPV?’

^b^Model controlled for age, region of residence, education and income

^c^Model controlled for age, region of residence and SES

^d^Continuous variable

^e^Reference: western Germany

^f^Reference: basic education

^g^Reference: low income

^h^Reference: low SES

^j^OR: Odds Ratio

^k^CI: Confidence Interval

Because the SES variable included the education and income variables as two of its components, two different multivariable logistic regression analyses were performed, one including education and income, and the other including SES. The multivariable logistic regression model including age, area of residence, education and income showed that girls whose mother had high education (OR: 1.5, 95 % CI 1.0–2.3) were more likely to have already been vaccinated compared to those whose mother had a basic education (Table 3). A similar statistically significant effect was found when comparing daughters of mothers with medium education (OR: 1.5, 95 % CI 1.1–2.1) to those with basic education. When SES was included in the multivariable model instead of education and income, girls of mothers with medium SES (OR: 1.5, 95 % CI 1.0–2.4) were more likely to have been vaccinated compared to girls of mothers with low SES. Age of the girl remained a statistically significant predictor in both multivariable models, with an increased likelihood of being vaccinated per year of increased age (OR: 1.6, 95 % CI 1.5–1.7).

## Discussion

To our knowledge, this is the first study on the influence of maternal education, household income and SES on the actual uptake of the HPV vaccine among girls in Germany during the first year of vaccine availability. Previous international studies have examined the influence of various factors on the intention to immunise a child [16, 21–23]. Studies have also been conducted on factors influencing actual vaccine uptake, although most of these studies were conducted in the United States [11].

The uptake of the HPV vaccine in this German population of girls aged nine to 17 years was 17.4 %. Although at the time of the survey the STIKO recommended immunising girls aged 12–17 against HPV [7], only 23.8 % of girls in this age group reported that they had already been vaccinated during the first year of vaccine availability. Across all ages, our results showed that older girls were more likely than younger girls to have been vaccinated, and that girls whose mothers had a high education, medium education or medium SES were more likely to have already been vaccinated than those whose mothers had a basic education or low SES. Region of residence and income were not found to have a statistically significant effect in the multivariable models.

A German survey conducted between 2009 and 2011 reported that 39.5 % of girls aged 14–17 had received the three-dose course of the HPV vaccine [24], while another German study conducted in 2010 reported that 49 % of women aged 18–20 had been vaccinated [25]. Similarly to Germany, France offers vaccination on request and achieved only 24 % coverage with three doses among 14 year-old girls in 2008 [26]. In the United States in 2007, it was estimated that 25.1 % of girls aged 13 to 17 had received at least one of the three required doses, and a fourth of those had completed the full vaccine course [27]. The 2007 California Health Interview Survey (CHIS) found that 26 % of girls aged between 13 and 17 had received at least one dose of the HPV vaccine, and 11 % had completed the series of three doses [28]. The Californian study also asked teenagers about their intention to be vaccinated, and 76 % of non-vaccinated 13 to 17 year-olds indicated that they were interested in being vaccinated [28]. These data are similar to our findings, where 74.5 % of girls aged nine to 17 who had not yet been vaccinated were open to the idea or had plans to do so. Vaccination in the United States has improved since the vaccine was first introduced, with the Centers for Disease Control and Prevention (CDC) reporting that in 2010, 32 % of girls aged between 13 and 17 had received three doses of the vaccine [29].

Australia, Scotland and England, in contrast to Germany, the United States and France, have implemented nationwide organised, school-based HPV immunisation programmes and therefore have direct access to the target population. Australia, which implemented a school-based programme in 2007, had achieved an average three-dose coverage for girls aged 12 of between 64 and 80 % by 2009 [30]. The National Health Services of Scotland reported that, after implementing a school-based vaccination programme, 88 % of girls in the second, fifth and sixth years of secondary school (approximate ages 13, 16 and 17) received the first two vaccine doses between September 2008 and February 2009 [31]. September 2011 figures from Scotland reported that 92 % of girls progressing through the system had received a first dose, with 81 % completing the course [32]. Similarly, within a school-based immunisation programme, 70.6 % of 2 817 grade eight girls at secondary schools in Manchester, England had received the first dose of the HPV vaccine, and 68.5 % had also received the second [33]. Three-dose coverage in England was reported to be 76 % in 2010 [34]. The Netherlands, which has a population-based, non-school-based HPV vaccination programme, reported 49.9 % coverage for the first dose among girls aged 13–16 after starting their programme in March 2009 [10]. In 2011, coverage in the Netherlands was reported to be 58 % among 12–13 year-old girls [26].

Multivariable models in our study showed that girls whose mothers had high education (OR: 1.5, 95 % CI 1.0–2.3) were more likely to have already been vaccinated compared to those whose mothers had basic education. Rosenthal and colleagues, however, found no significant association between the mother’s education and the daughter’s vaccination uptake in a study conducted in the United States [35]. Other studies in the United States and Canada investigated the association of parental (mothers and fathers) education on HPV vaccine uptake, with inconsistent findings [23, 36, 37]. Brewer and colleagues found higher vaccine uptake in girls of parents with a college education compared to a high school education [37]. Chao and colleagues reported that higher neighbourhood education level was positively associated with vaccine regimen completion in California [38].

Our findings showed that girls of mothers with medium SES (OR: 1.5, 95 % CI 1.0–2.4) were more likely to have been vaccinated compared to girls of mothers with low SES. Findings from another German study revealed an increased likelihood of being vaccinated among girls from medium SES, compared to high SES, families (OR: 1.9, 95 % CI 1.3–2.8) [24].

Low vaccine coverage potentially limits the impact of the HPV vaccine on the reduction of cervical cancer incidence and mortality [39]. Several studies have concluded the vaccine to be cost-effective, but these studies assumed vaccine coverage rates to be at least 50 % or higher [40, 41]. Part of maximising the cost-effectiveness of the HPV vaccine therefore involves achieving high coverage in young adolescent girls [42].

Limitations of our study include using self-reported data. It could be argued that very young girls may have had difficulty understanding the question relating to HPV vaccination. However, the girls filled out the questionnaire within their home environment, with the consent of their parents, and could have asked their parents to clarify any parts of the question which may have been unclear. No data were collected regarding how many of the girls who indicated being vaccinated had actually completed the full course of three vaccination doses. In addition, information regarding reasons why a girl was not vaccinated was not available. Although almost 2 000 girls aged nine to 17 filled out the questionnaire, the response rate of 40.2 % was relatively low. In order to determine the potential selection bias introduced by this fairly low response rate, we assessed households with a child (boy or girl) aged younger than 18 years based on 2007 data from the Federal Statistical Office [43] and found a very similar distribution of household income when comparing the households included in our analysis with this subgroup in the German population. A total of 79 % of households included in our analysis had an income of 1500 € or more, compared to 81 % of households with a child aged younger than 18 years in the German population as a whole. We therefore did not consider weighting to be necessary in our analyses. A further limitation is that our data only allowed assessment of the mother’s and not the father’s SES and education. Nevertheless, the ‘mother’s SES’ variable includes ‘household income’ and in households where there was a father, this variable would also have taken the father’s income into account. Although the role of the mother is of primary importance in terms of communicating HPV-related issues with the daughter, the father may have also played an important role in terms of decision-making within the household [44]. It is important to note that the HPV vaccine had only been available in Germany for approximately one year when this survey was conducted.

## Conclusions

Although vaccination rates in Germany have increased over the past few years, the rates remain low. Other countries with organised, school-based programmes introduced the vaccine at approximately the same time and have had much higher uptake rates during the first year following vaccination introduction, as well as in subsequent years. If higher vaccination rates are to be achieved in Germany, an organised HPV vaccination programme, ideally school-based, will need to be introduced. Additionally, reasons why girls or their parents might decide against vaccination should be investigated. Consideration should be given to strategies aimed at reaching the population groups identified in this study that were less likely to be vaccinated, such as younger girls and girls whose mothers have lower levels of education and SES. High vaccination uptake is necessary to reduce the incidence of cervical cancer, and organised vaccination programmes would help to achieve this goal.

## Abbreviations

CDC: Centers for Disease Control and Prevention
CHIS: California health interview survey
CI: Confidence interval
EMA: European Medicines Agency
FDA: U.S. Food and Drug Administration
HPV: Human papillomavirus
OR: Odds ratio
SES: Socio-economic status
STIKO: German Standing Committee on Vaccination
